# The dynamic strategy shifting task: Optimisation of an operant task for assessing cognitive flexibility in rats

**DOI:** 10.3389/fpsyt.2024.1303728

**Published:** 2024-06-28

**Authors:** Jonathan Martin Flintoff, Suzy Alexander, James Paul Kesby, Thomas Henry Burne

**Affiliations:** ^1^ Queensland Brain Institute, The University of Queensland, St Lucia, QLD, Australia; ^2^ Queensland Centre for Mental Health Research, The Park Centre for Mental Health, Wacol, QLD, Australia

**Keywords:** cognitive flexibility, strategy-shifting, operant tasks, neuropsychiatric disease, translational research

## Abstract

**Introduction:**

Although schizophrenia is associated with a broad range of symptoms including hallucinations, delusions, and reduced motivation, measures of cognitive dysfunction, including cognitive flexibility and executive function, are the strongest predictors of functional outcomes. Antipsychotic medications are useful for reducing psychotic symptoms, but they are ineffective at improving cognitive deficits. Despite extensive investment by industry, the transition from preclinical to clinical trials has not been successful for developing precognitive medications for individuals with schizophrenia. Here, we describe the optimisation of a novel dynamic strategy shifting task (DSST) using standard operant chambers to investigate the optimal stimuli required to limit the extensive training times required in previous tasks.

**Methods:**

We determined that optimal learning by male and female Sprague Dawley rats for the flexibility task incorporated dynamic strategy shifts between spatial rules, such as following a visual cue or responding at one location, and non-spatial rules, such as responding to a central visual or auditory cue. A minimum of 6 correct consecutive responses were required to make a within-session change in the behavioural strategies. As a proof of concept, we trained and tested 84 Sprague Dawley rats on the DSST, and then assessed their cognitive flexibility using a within-subject design after an acute dose of ketamine (0, 3, 10 mg/kg). Rats made fewer premature and more perseverant responses to initiate a trial following ketamine. The effects of ketamine on trials to criterion was dependent on the rule.

**Results:**

Ketamine induced a significant improvement on the reversal of a non-spatial visual discrimination rule. There was no significant effect of ketamine on the spatial visual or response discrimination rules.

**Discussion:**

The DSST is a novel assay for studying distinct forms of cognitive flexibility and offers a rapid and adaptable means of assessing the ability to shift between increasingly challenging rule conditions. The DSST has potential utility in advancing our understanding of cognitive processes and the underlying neurobiological mechanisms related to flexibility in neuropsychiatric and neurological conditions where executive dysfunctions occur.>

## 1 Introduction

Schizophrenia is a debilitating neuropsychiatric illness with a worldwide prevalence of 0.7% and typically has an onset in young adults aged 16–26 years (1). It is characterised by positive symptoms that include visual and/or auditory hallucinations (2), negative symptoms such as flattened affect (3), and cognitive symptoms that impair domains of cognition necessary for daily living (4, 5). Individuals with schizophrenia often show cognitive impairments in verbal learning and memory, visual learning and memory, reasoning and problem-solving, attention, and processing speed (6). Cognitive symptoms have a particularly strong association with reduced quality of life (7). Current first- and second-generation antipsychotics have limited efficacy in improving cognition (8) and have major side effects that increase mortality (9). Treatment strategies capable of targeting specific areas of cognitive impairment and those that target a common pathophysiological mechanism implicated in cognitive impairment are necessary for improving treatment options (10).

Cognitive flexibility and behavioural flexibility refer to a set of cognitive processes that allow us to monitor and maintain behaviour when required, but flexibly adapt behaviour when faced with changing environmental contingencies (11, 12). In this way, cognitive flexibility is fundamental in optimising adaptability, rather than automaticity in behaviour. Cognitive flexibility can be comprised of attentional set-shifting (also termed set-shifting), task-shifting, and reversal learning (13). Attentional set-shifting involves adapting attention to different rules or stimuli, while task-shifting requires shifting attention between a task with distinct goals or requirements. Reversal learning focuses on modifying strategies to achieve the same goal. These separate aspects of cognitive flexibility require an organism to have a representation of a goal (rule generation), maintenance of behaviour to achieve that goal (rule selection), and the ability to disengage from a previous set of actions when encountering changing environmental contingencies (14). Cognitive flexibility impairments within psychiatric disorders can be characterised by behaviours that are highly resistant to change and result in maintaining (perseverating) a maladaptive behaviour (15). Such maladaptive behaviours arise when there is a failure to modulate response selection when required. Understanding the underlying neurobiology in humans and animals is imperative if we are to understand how it is altered in neuropsychiatric disorders.

Recent trends in animal research have focused on the use of low, moderate, and high doses of ketamine to model and treat neuropsychiatric disorders. Low doses of ketamine rapidly alleviate depressive symptoms in humans (16). Animal work continues to elucidate its mechanisms of action that include N-methyl-D-aspartate receptor (NMDAR) channel blockade (17), release of dopamine and noradrenaline (18, 19), and activation of AMPA receptors (20). These actions lead to the activation of downstream signalling pathways, such as the mTOR pathway, which promote synaptic plasticity and neurogenesis (21, 22). Importantly, the effects of ketamine are dose dependent. Lower doses may have a biphasic effect whereby short-term dysfunction and longer-term improvement in memory are present (23), whereas higher doses have been shown to impair memory consolidation (24). Such doses induce pronounced NMDA receptor hypofunction, leading to widespread cortical dysfunction, disrupted gamma oscillations, and neurotransmitter imbalances, which collectively result in cognitive impairments and psychotic-like behaviours (25–27). This model helps researchers study the underlying mechanisms of schizophrenia and evaluate potential therapeutic interventions (25, 27, 28). The dual use of ketamine in animal models and human therapeutics underscores its complex pharmacodynamics and the importance of considering dose when interpreting its effects in behavioural research.

Numerous behavioural assays have been developed in rodents that probe cognitive flexibility through attentional set-shifting and strategy shifting. The widely established attentional set-shifting task (ASST) requires rats to discriminate between two stimuli that differ based on two dimensions, odour and digging medium (29). The task comprises seven stages where rats learn to discriminate the bowls and dig in one of two bowls based on a food reward associated with the relevant stimulus dimension and at the extradimensional (ED) stage of the task, rats are required to overcome the attentional set formed through learning stimulus-reward pairings in one particular stimulus dimension (intradimensional; ID). Importantly, at the ED stage, rats must overcome an attentional set, and lesion studies have shown that this process requires the medial prefrontal cortex (mPFC) (29). The ASST has been useful in detecting frontal-executive dysfunction in models of lead exposure (30) and instrumental in establishing pharmacological models of schizophrenia that have advanced our understanding of how distinct neurotransmitter systems contribute to cognitive flexibility (31). While the ID/ED ASST is a robust task effective at measuring the cognitive flexibility involved in attentional set-shifting behaviour (32), much of the training and testing is manual-based which limits the throughput of the task. Similar to the Cambridge Neuropsychological Testing Automated Battery (CANTAB) ID/ED task, the touchscreen operant platform allows for a wide range of visual discriminations (33) using visual cues whereby the rodent must select the appropriate stimulus on the screen to receive a food reward. While having high comparability to human tasks such as the CANTAB ID/ED, one major hurdle to implementing this type of behavioural assay is the number of training sessions required (34). A more recent ID/ED task was developed by Scheggia and Papaleo (35) which provides a completely automated setup of three dimensions of stimuli, including texture, odour, and light stimuli. This task improves on previous paradigms as the two-chambered set-up allows automatic and continuous stimuli changes without interfering with the mouse and has been validated using several genetic models of schizophrenia but has not been widely adapted for rats. Alternatively, several tasks have been developed that explore strategy shifting behaviour. Brady and Floresco (36) developed a strategy shifting task that occurs within an operant conditioning chamber that has two levers, two cue lights and a central reward receptacle. In this task, the subject must learn to press a lever to obtain a pellet reward from a central receptacle. Here there is a strategy-shift between following a cue light (visual cue discrimination) and responding at the appropriate side to then responding only on one side (response discrimination). Although the rat does not initiate the trial, and each strategy is run in separate sessions, the collection of data using this task is rapid, automated, and both rats and mice can learn the task (37).

The aim of this study was to develop a dynamic strategy shifting task (DSST), which is best conceptualised as an extension of the strategy shifting task developed by Floresco and colleagues (37). However, we also wanted to incorporate multiple strategy shifts of increasing difficulty within a session. The DSST was created to replicate the features of human neuropsychological tasks that assess executive function such as the Wisconsin Card Sorting Test (38). Namely, this task examines a rat’s ability to shift between different strategies (attending to a visual cue, responding at one location). One of the primary findings of this study was the critical role of the mPFC in facilitating a shift in attention from a cue light discrimination to an egocentric response. Importantly, it was found that an increase in difficulty in the shift between rules was linked with a decrease in performance when the mPFC was lesioned (37). While the mPFC is central to mediating cognitive flexibility, connections between the mPFC and nucleus accumbens shell, contribute to maintaining new strategies (39). Additionally, input from the mediodorsal thalamus to the nucleus accumbens core aid in eliminating inappropriate strategies (39). When perseveration has ceased, connections between the nucleus accumbens shell and dorsal medial striatum mediate the maintenance of a newly acquired strategy (40). Evidently, strategy selection, suppression of prior responses, and maintenance of a successful strategy rely on the successful integration of dissociable aspects of flexible behaviour. Tasks that contribute to parcellating different forms of cognitive flexibility are essential to understanding how this executive function is altered in neuropsychiatric diseases.

The DSST was also designed to allow investigation of performance across stages that may require increasing engagement from the mPFC as well as assessing performance across multiple stimulus modalities relevant to deficits seen in neuropsychiatric diseases (41). Each strategy shift within the DSST requires action selection, suppression of prior responses, and maintenance of strategy. However, the demands of each stage were designed to increasingly challenge cognitive load. Once rats were trained to press a lever on a continuous reinforcement schedule they were trained in a simple visual cue discrimination task (referred to as the visual cued task; VCT), in which the rat had to press a lever below an illuminated cue light. The next task was a response discrimination, in which the rat had to respond to the lever on one side, and ignore the cue light (fixed location task; FLT). We next exposed rats to a non-spatial visual discrimination task (referred to as the visual continuous detection task; VCDT) in which the rat had to learn to respond on one lever when a central cue light came on. Finally, rats were required to transition to a non-spatial auditory cue using an auditory continuous detection task (ACDT). The ACDT was incorporated as an orienting behaviour towards a previously untrained sensory modality as used in human tasks (42). Importantly, alterations in task order or stage-specific manipulations, including the possibility of reversals and distractors, provide a framework to dissect multiple forms of cognitive flexibility, offering valuable insights into the neural and pharmacological substrates underlying these processes.

To develop the DSST, a series of experiments were undertaken to determine whether Sprague Dawley rats were able to rapidly learn to discriminate single images and adapt to changing lever-image associations. We then explored each measure on the DSST and how it predicted performance. After the development of the DSST, several manipulations were tested to optimise task parameters. Sex differences in performance were explored in addition to optimal stimuli that could be used by Sprague Dawley rats. The addition of an automated rule change was also investigated to determine the highest performance capabilities of rats rapidly switching between a cue-based rule to a fixed location rule. Finally, as a proof of concept, we trained and tested 84 rats within 14 days and then administered an acute dose of the NMDAR antagonist ketamine using a within-subject Latin-square design. This drug has been used extensively to model cognitive deficits related to mPFC disruption in rodents (43). In this experiment, we incorporated rule changes across different modalities (visual to auditory) to examine an effect of ketamine during heightened task difficulty. Overall, the results from this study have allowed optimisation of the design features implemented in the DSST to measure cognitive flexibility in rats, and suggest that the DSST incorporates novel features that are not present in existing rodent tasks of cognitive flexibility.

## 2 Materials and methods

### 2.1 Animals and housing

Male and female Sprague Dawley rats (N=150, ARC, WA) aged 8 weeks were housed in wire-top cages. Rats in Cohort 1 (n=16) were used for the image discrimination protocol, rats in Cohort 2 (n=16) were used for the dynamic reversal protocol, rats in Cohort 3 (n=34) were used for the optimised DSST protocol, and rats in Cohorts 4 (n=48) and 5 (n=36) were used for the ketamine manipulation. Each cage contained aspen chip bedding, holed housing, hammocks, nesting and a wood chew. Rats were housed at 21 ± 2°C and 60% humidity. A 12-hour standard light cycle was used (lights on at 8:00 AM). Due to an electrical fault in the animal facility, some of the rats used in the ketamine manipulation (Cohort 5; n=32) were exposed to constant light for the duration of the experiment. Rats were weighed daily and ear-notched for identification. At 10 weeks of age, rats were food restricted to 90% of their free-feeding body weight and with ad libitum access to water. The rats were trained and fed at the same time each day, and within each manipulation, the experimental unit was a single rat. All procedures were performed with approval from The University of Queensland Animal Ethics Committee, under the guidelines of the National Health and Medical Research Council of Australia.

### 2.2 Apparatus

**Figure 1:**
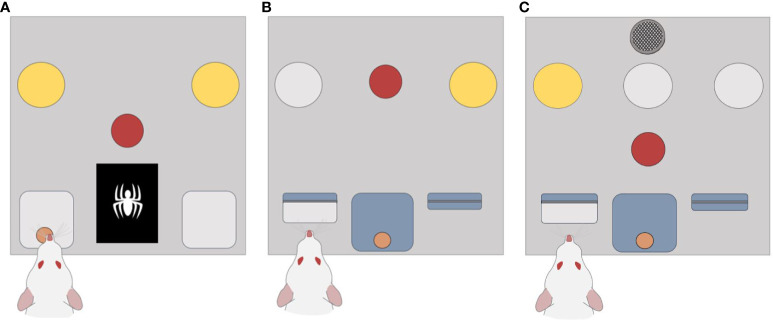
Configurations used for optimising the DSST. **(A)** Schematic design of the chambers used in the visual discrimination manipulation. A central nose poke port was required to initiate each trial (shown as the red circle). Rats were then required to observe the central display for an image (white spider) and respond in a head entry receptacle either side (grey square) to receive a pellet reward. **(B)** Shows the schematic design of chambers used during the dynamic reversal manipulation. Each trial started with a response in the central nose poke that was followed by presentation of stimulus. The subject was then required to respond by pressing a lever. **(C)** For the optimised DSST configuration the front panel consisted of a speaker, 3 stimulus cue lights, an illuminated nose poke, two retractable levers, and a central reward magazine.

Experiments were conducted using 12 Med Associates operant chambers for rats (Med Associates Inc., St Albans, VT, USA). Each chamber was in a sound-attenuating cage with a ventilation fan and overhead camera (Revotech, Los Angeles, USA). During initial stimulus optimisation, an LCD screen (ENV-132M, 5.0 x 3.8 cm) was used to examine complex image discrimination ability. Subsequently, each operant box was constructed with a solid clear polycarbonate roof and door, aluminium channels to hold modular components from Med Associates including: a central house light (ENV-215M-LED, 55 lx) located on the back wall, two retractable levers (ENV-112CM) next to a central reward receptacle (ENV-254-CB) on the front wall, 3 cue lights (ENV-221M, 40 lx) located on the left, middle, and right central above the left retractable lever, central reward receptacle, and right retractable lever respectively. A central nose poke port (ENV-275-NPP) on the front panel was used to initiate trials and a Sonalert module (ENV-223AM) used to generate a tone cue (85 dB). For reward delivery there was a motorised pedestal mount pellet dispenser (ENV-203M-45) dispensing grain pellets (45 mg, ASF0165, Able scientific, Auckland) into the central reward receptacle. Protocols were written using MedState Notation and Med-PC for Windows software (Med Associates Inc., St. Albans, VT, USA) was used for chamber operation and data collection. Examples of the main wall are shown for the image discrimination protocol (**Figure 1A**), the dynamic reversal protocol (**Figure 1B**), and the optimised DSST protocol (**Figure 1C**). A flowchart of the optimised DSST protocol is shown in **Supplementary Figure 1**.

### 2.3 Operant training procedure for image discrimination and dynamic reversal protocol

In Stage 1, rats were trained to collect pellets from the magazine after making a nose poke entry. Each head entry resulted in a reward being collected with a maximum of 50 pellets collected in each reward magazine over two consecutive days. Stage 2 required rats to learn to make a head entry into the central nose poke to initiate trials. After a trial was initiated, an image or a cue light was presented, and the correct head entry magazine was illuminated, with a head entry required to receive a pellet reward. To move on to Stage 3 of training, 80 correct trials were required and there was no consequence for an incorrect response. Stage 4 of training required the rats to self-initiate the trial after which time the cue light stimulus was presented for 1 second after which time both reward receptacles were illuminated. The rats were required to make a head entry into the correct reward receptacle to receive a pellet reward. A limited hold duration of 10 seconds was present in which the rat was required to make a response. If no response was made, an omission was recorded and the trial ended. Rats were required to achieve 80% correct head entries in this learning stage. If an incorrect response was recorded, a time-out phase of 5 seconds was initiated. A list of outcome measures derived from this task is shown in **Table 1**.

**Table 1:** Potential outcome measures on the DSST.

Measure	Calculation
**% Accuracy**	Σ correct trials/correct trials + incorrect trials *100
**Correct trials**	Σ correct trials
**Incorrect trials**	Σ incorrect trials
**Trials to criterion**	Σ trials before the rat achieves the required consecutive correct response number
**Premature head entries**	Σ head entries made prior to making a choice
**Perseverant head entries**	Σ head entries made after making a choice
**Perseverative error**	Σ incorrect lever presses during the first 6 trials after a strategy shift.
**Omission trials**	Σ initiated trials where the rat fails to respond within 10 seconds
**Average trial duration**	time (s) taken to complete a single trial
**Win-stay percentage**	Win-stay/win-stay + win-shift trials * 100
**Lose-shift percentage**	Lose-shift/lose-shift + lose-stay trials * 100

#### 2.3.1 Stimulus optimisation

After establishing the training protocol, experimental manipulations were conducted to find the most suitable stimuli to design the strategy shifting task with n=16 Sprague Dawley rats (n=8 males, n=8 females; Cohort 1). This sample size was chosen to produce preliminary data to assess the image discrimination capabilities of Sprague-Dawley rats. A within-subjects design was used to assess each rat’s performance across each image manipulation. The aim was to assess the performance of a group of male and female Sprague Dawley rats in discriminating images to obtain a reward. Several pairs of images were trialled. With each image pair, the first day consisted of a visual cue task (VCT) whereby rats responded via a head entry into a left or right magazine under the same location as a cue light. On subsequent days, one of two images was presented to the rat per trial. The rat had to learn to associate each image with a left or right head-entry response (image-side pairing was randomised). The primary readout for these manipulations was % accuracy to determine if the rats were responding at an above chance level. A threshold of 80% accuracy was selected to compare the performance of rats using the current setup with acquisition curves of rats tested on similar visual discrimination tasks (33). One image was presented in any given trial with one image being associated with a correct left or right head entry response to receiving a reward. In the first experimental manipulation, rats were presented with four image pairs: left vs. right positioned squares, spider vs. aeroplane, grey vs. black, and vertical vs. horizontal Gabor patch.

#### 2.3.2 Dynamic reversal manipulation

This manipulation investigated the performance of 16 Sprague Dawley rats (n=8 males, n=8 females; Cohort 2) shifting between the VCT whereby the rat was required to respond underneath the illuminated cue light to a fixed location task (FLT) where the rat was required to respond only on one side. The first aim of this manipulation was to determine how many trials it takes for rats to make a shift between these strategies and whether sex differences in performance were apparent. Rats were trained to follow a presented cue light signalling a reward in either the right or left reward receptacle. Three reversal manipulations were conducted whereby rats were required to shift from following the cue light to only making a response in a fixed reward location. This reward location (left or right) was randomised throughout the cohort. To observe how many trials were needed for the rats to reach a threshold of 80% accuracy on each rule, rule shifts were enforced after 60, 40, 30, and 20 trials. These manipulations were carried out on separate days, each after the baseline task had been performed.

### 2.4 DSST protocol

**Figure 2:**
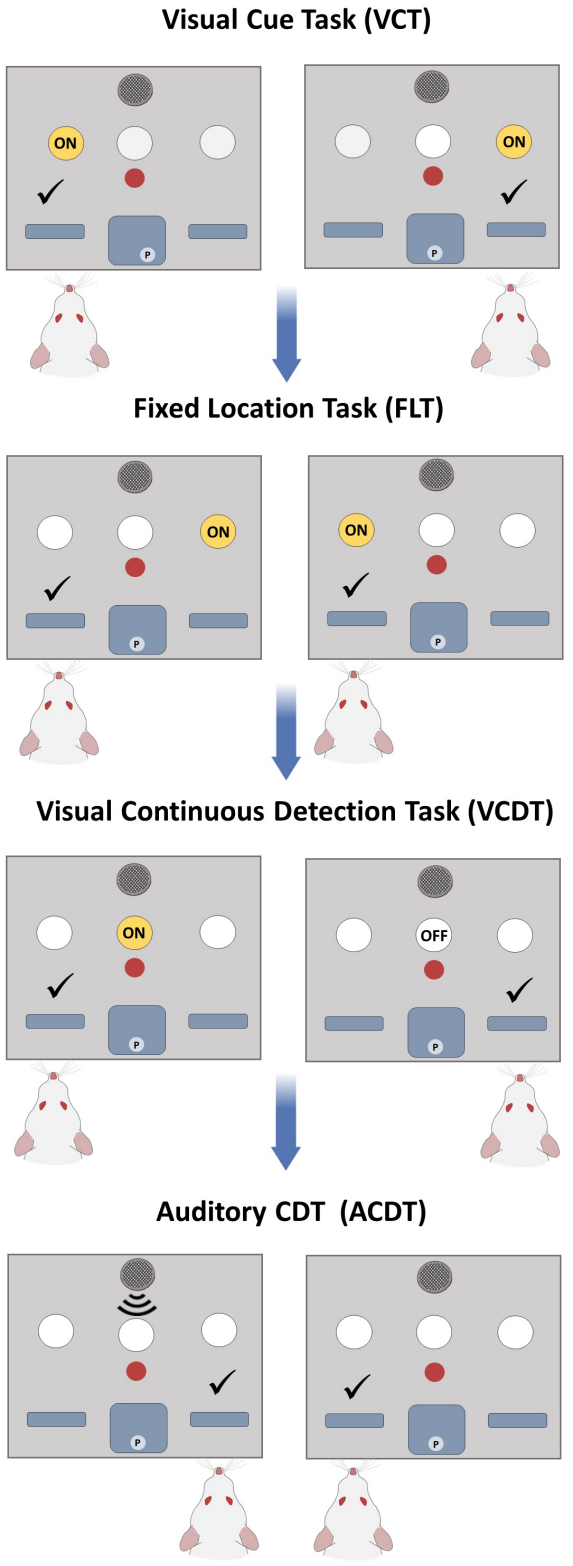
Optimised DSST Protocol. During the testing phase, rats progressed through a series of strategy shifts. Rats had to first learn to follow a cue light and respond below the location of the visual cue (VCT), then respond only at one location (FLT). Subsequently they learnt a stimulus-response pairing during signal trials by attending to a central cue light (VCDT) and learn the reverse stimulus-response pairing during signal trials using an auditory tone ACDT. Reversals could be incorporated after FLT, VCDT or ACDT stages.

We next tested n=34 Sprague Dawley rats on the optimised DSST protocol (Cohort 3). In brief, the training phase consisted of magazine training whereby after initiation of the trial, ten pellets were dispensed, and rats were required to collect 100 pellets from the central reward magazine (Stage 1). Then the rat was required to initiate trials by responding in the central nose poke (Stage 2). Subsequently, levers were coated with a paste containing the reward pellets and the rat was required to respond by pressing the lever to receive a reward (Stage 3). Finally, rats were required to respond within ten seconds of lever presentation before retraction of the levers (Stage 4). During the testing phase, rats were given 60 minutes per session and an additional 30 minutes when the rat obtained a consecutive correct response (CCR) rate of 10 (we subsequently reduced to the CCR to 6; see below; See **Table 2** for task stages). All rats began on the VCT (left and right cue trials were counterbalanced) and once achieving a consecutive correct response rate of 10 moved sequentially to the FLT. On the FLT trials, the number of rats randomised to a fixed right or left reward response was counterbalanced. Rats then progressed to the VCDT, ACDT, and finally to the ACDT reversal (ACDTR). During each of these stages the number of rats in the signal = left response and non-signal = right response was counterbalanced. Signal and non-signal trials were presented in equal numbers and only 4 trials of the same type were possible. A limited hold period of 10 seconds was used during testing, and a timeout period of 5 seconds was initiated after an incorrect response. Omissions did not reset the correct response count. Perseverative errors were calculated if the rat made an incorrect response that corresponded to the previous rule. Win-stay and lose-shift responses were calculated based on the strategy required during each stage i.e. win-stay on the VCT is a response below the cue light and win-stay on the FLT is a response at the same location. Each of the rules (VCT, FLT, VCDT and ACDT) is shown in **Figure 2**.

**Table 2:** Stages on the optimised DSST.

Stage	Trials	Session (min)	Dimension	Limited Hold (s)	Timeout (s)	Criterion
1. Free pellets	100	30	N/A	N/A	10	100 pellets
2. Nose poke initiation	100	30	N/A	N/A	10	100 pellets in 30 minutes
3. Lever press	100	30	N/A	N/A	10	100 pellets in .30 minutes
4. Timed lever press	120	30	N/A	10	10	120 trials in 30 minutes
VCT: Visual Cued Task	<120	60	Visual	10	5	6 CCR
FLT: Fixed Location Task	<120	60	Spatial	10	5	6 CCR
FLT-reversal: Fixed Location Task reversal	<120	60	Spatial	10	5	6 CCR
VCDT: Visual Continuous Detection Task	<120	60	Non-spatial Visual	10	5	6 CCR
VCDT-reversal: Visual Continuous Detection Task reversal	<120	60	Non-spatial Visual	10	5	6 CCR
ACDT: Auditory Continuous Detection Task	<120	60	Non-spatial Auditory	10	5	6 CCR
ACDT-reversal: Auditory Continuous Detection Task reversal	<120	60	Non-spatial Auditory	10	5	6 CCR

### 2.5 Acute ketamine manipulation

As a proof of concept for high throughput screening of pharmacological agents, we tested 84 Sprague Dawley rats across two separate cohorts (Cohort 4: n=24 females, n=24 males. Cohort 5; n=18 females, n=18 males) in a within-subject Latin square design in response to ketamine (0, 3 or 10 mg/kg). Rats were randomised to receive each dose 30 minutes before testing, such that all rats received every dose over 3 consecutive days. Rats were initially food restricted to 85% of their free feeding body weight and then trained using Stages 1–4 as outlined above. Once they had passed Stage 4 the rats were exposed to each of the rules in the DSST. If a rat completed a CCR count of 6 for a particular rule, then it moved onto the next rule. A CCR count of 6 was chosen to ensure comparability with previous cognitive flexibility paradigms (29) and serve as a stringent criterion that reduces rats achieving a rule-shift by chance but enables high-throughput assessment of performance. Each rat was required to pass each stage in the primary exposure (VCT, FLT, FLT-reversal, VCDT, ACDT), and rats were tested over multiple session until they reached criteria. The average number of trials predicted to reach a CCR of 6 is 126. Once they had reached criteria for all rules in the primary exposure, they were allocated to a drug dose order group. Rats were tested over 3 consecutive days. On each day they were injected once s.c. and 30 minutes later placed in the operant chambers and given a secondary exposure across 2 sessions separated by 4 hours. The order of rules was VCT, FLT, FLT-reversal, VCDT, VCDT-reversal, ACDT, ACDT-reversal. The session ended if 120 trials were completed without passing a rule or if there was inactivity for >5 minutes. The second session was used if rats did not complete all rules in the first session. Under these conditions (a limit of 240 trials to achieve a CCR of 6) the average number of trials predicted to reach 6 CCR is 88. Analysis was carried out by a researcher blind to treatment group at the end of the experiment. A counterbalanced Latin square design was used to ensure treatments and rule exposures occurred equally on each day, and in each operant box during the experiment. Randomised treatment allocation was performed using the =RAND() function in excel.

### 2.6 Statistical analysis

Descriptive statistics were used to calculate the mean, standard error, minimum and maximum values for each outcome measure. Changes in each outcome measure during the VCT and FLT were analysed with a one-way ANOVA. If the data did not meet the assumption of normality as indicated by a Kolmogorov-Smirnov test, a nonparametric test such as Welch’s ANOVA was used. To further investigate main effects, Bonferroni correction was used to explore multiple comparisons in SPSS (version 25). For the ketamine experiment, rats were required to successfully complete training stages of the task and the first exposure of each rule prior to drug treatment. The data from n=3 rats were excluded from the ketamine manipulation due to incorrect dosing schedule. The primary measure was the trials to criterion for each rule, but we also analysed premature and perseverant responses to initiate a trial, the number of errors in the first 6 trails of a new rule, and the response time. Prior to analysis of the main effects of Dose (0, 3, 10 mg/kg) and Sex (female or male), we assessed the effect of Testing Day (1, 2 or 3) to assess test order effects, and the effect of Cohort (5 or 6), due to the altered lighting conditions in the animal facility. Where possible we analysed data with repeated measures ANOVA. However, due to missing trials to criteria data for some of the rules in the ketamine experiment (VCDT-reversal and ACDT), hazard ratios (HRs) and 95% confidence intervals (CIs) were computed with a Cox regression analysis, where HRs <1 correspond to a lower score, and HRs >1 correspond to a higher score after ketamine compared with saline treatment. Significance was set at a threshold of *p*<0.05. Effect sizes are reported as partial eta-squared values (η_p_^2^) for ANOVAs, and Cohen’s d for t-tests. Graphs were made using GraphPad Prism version 10.

## 3 Results

### 3.1 Image discrimination manipulation

**Figure 3:**
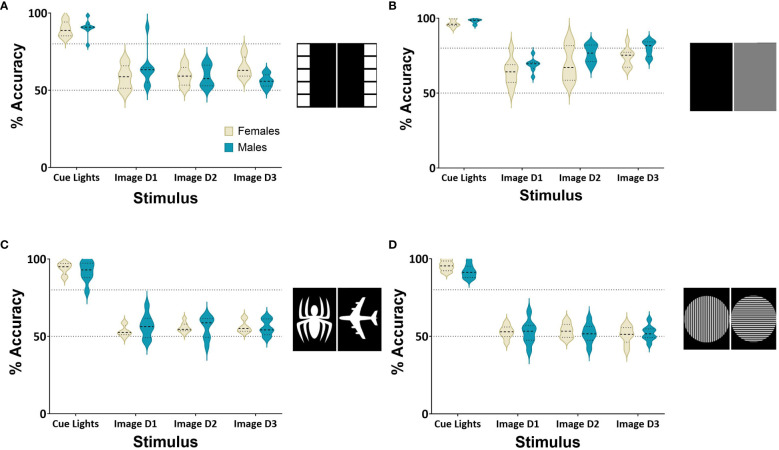
Response accuracy during image discrimination. Performance accuracy in the image discrimination task on baseline task and 3 days of image discrimination trials. **(A)** Shows performance accuracy on 1 day of the VCCT compared to 3 days of the square image pair. **(B)** Shows performance accuracy on 1 day of the baseline compared to 3 days of the grey and black image pair. **(C)** Shows performance accuracy on 1 day of the baseline compared to 3 days of the spider and aeroplane image pair. **(D)** shows performance accuracy on 1 day of the baseline compared to 3 days of the vertical and horizontal Gabor patches. Cohort consisted of *n*=8 males and *n*=8 females. The criteria for successful acquisition of image discrimination was determined by achieving at least 80% accuracy in a session.

Male and female rats (*n*=8 per group) were first required to complete training and learn to follow a cue light to receive rewards from either the right or left head entry receptacle. To compare performance, the image manipulation consisted of one day of the VCT, followed by three days of image manipulation training to assess whether they could achieve 80% accuracy. A repeated measures ANOVA indicated a significantly decreased performance during the square image discrimination task compared to the VCT (*F*_(3,42)_=86.63*, p*<0.001, η_p_^2^ = 0.76 **Figure 3A**). In the second manipulation, rats were required to discriminate between grey and black images over three sessions. Rats on this manipulation achieved 80% accuracy on Day 3, showing these rats could learn to discriminate based on luminance (**Figure 3B**). Over all days of the manipulations, a repeated measures ANOVA indicated an effect of Day on Performance (*F*_(3,42)_=124*, p*<0.01, η_p_^2^ = 0.75) demonstrating the performance decreased significantly after the VCT, but there was no effect of Sex (*F*_(1,14)_=3.35*, p*=0.08, η_p_^2^ = 0.03). Rats could not form response-reward associations with the Spider and Aeroplane images (**Figure 3C**), with a repeated measures ANOVA finding a significant decrease in Performance (*F*_(3,42)_=261*, p*<0.001, η_p_^2^ = 0.91), but no main effect of Sex (*F*_(1,14)_=0.02*, p*=0.88, η_p_^2^ = 0.001). The final image manipulation was conducted to observe whether rats could discriminate between a Gabor patch oriented either vertically or horizontally. Males and females performed significantly worse after the VCT, with a repeated measures ANOVA showing a main effect of Day (*F*_(3,42)_=249*, p*<0.01, η_p_^2^ = 0.93), but no main effect of Sex (*F*_(1,14)_=0.68*, p*=0.42, η_p_^2^ = 0.001) (see **Figure 3D**).

### 3.2 Dynamic reversal manipulation

**Figure 4:**
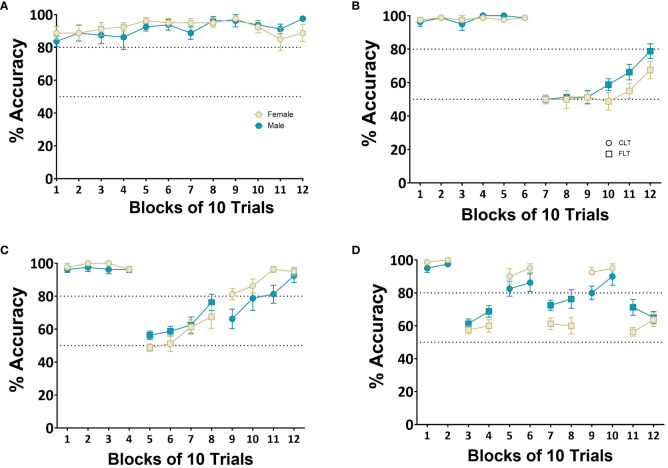
Dynamic reversal manipulation. Shows the dynamic switch protocol whereby rats (*n*=8 males, *n*=8 females) were required to switch between the two rules after a fixed number of trials. The first rule was the cue light task requiring rats to respond at the location of a cue light by making a head entry in the correct head entry receptacle. The second rule required rats to respond only at one location and withhold responding to the location of the cue light. Each block consisted of 10 trials. **(A)** performance on the VCT over 12 blocks. **(B)** Performance over 12 blocks with 60 trials on each rule. **(C)** Performance over 12 blocks of 40 trials per rule. **(D)** Performance over 12 blocks with 20 trials per rule. Successful acquisition of a rule was determined by achieving 80% accuracy.

Each block consisted of 10 trials where accuracy was calculated by dividing the number of correct responses by the total number of trials and multiplying this by 100. Rats were able to successfully acquire the baseline VCT with a repeated measures ANOVA showing no main effect of Block (*F*_(2,45)_=1.64*, p*=0.210, η_p_^2^ = 0.08) or Sex (*F*_(2,45)_=1.01*, p*=0.24, η_p_^2^ = 0.001) (**Figure 4A**). There was a main effect of Block when rats were required to shift to the FLT after 60 trials (*F*_(2,45)_=105.4*, p*<0.001, η_p_^2^ = 0.99) but no effect of Sex *(F*_(2,45)_ =0.35*, p*=0.96, η_p_^2^ = 0.72) (**Figure 4B**). A main effect of Block was also observed when rats were required to shift between rules after 40 trials (*F*_(2,45)_ =38.72*, p*<0.001, η_p_^2^ = 0.88) and again, no effect of Sex (*F*_(2,45)_=1.56*, p*=0.23, η_p_^2^ = 0.21) (**Figure 4C**). Finally, when rats were required to shift between rules after 20 trials, a main effect of Block was observed (*F*_(2,45)_=38.72*, p*<0.001, η_p_^2^ = 0.99) with no main effect of Sex (*F*_(2,45)_=0.72*, p*=0.41, η_p_^2^ = 0.05) (see **Figure 4D**). The rats demonstrated improved performance on both the VCT and FLT after each shift in rule, although lower accuracy was observed on the FLT compared with the VCT.

Rats trained on the screens required a mean number of 59 sessions (minimum 20, maximum 68) to reach criteria, compared to lever-trained rats that required 9 sessions (minimum 5, maximum 13). Given these data did not meet the assumption of normality according to a Kolmogorov-Smirnov test (*p*<0.0001), a Welch’s ANOVA test revealed a significant difference between training sessions with a main effect of Training Protocol on Sessions (*F*_(5,44)_=73.61*, p*<0.001, η_p_^2^ = 0.98). These results indicated that visual discrimination learning using screens was significantly slower than subsequent versions of the task where cue lights were used.

### 3.3 Baseline responses on the optimised DSST

**Figure 5:**
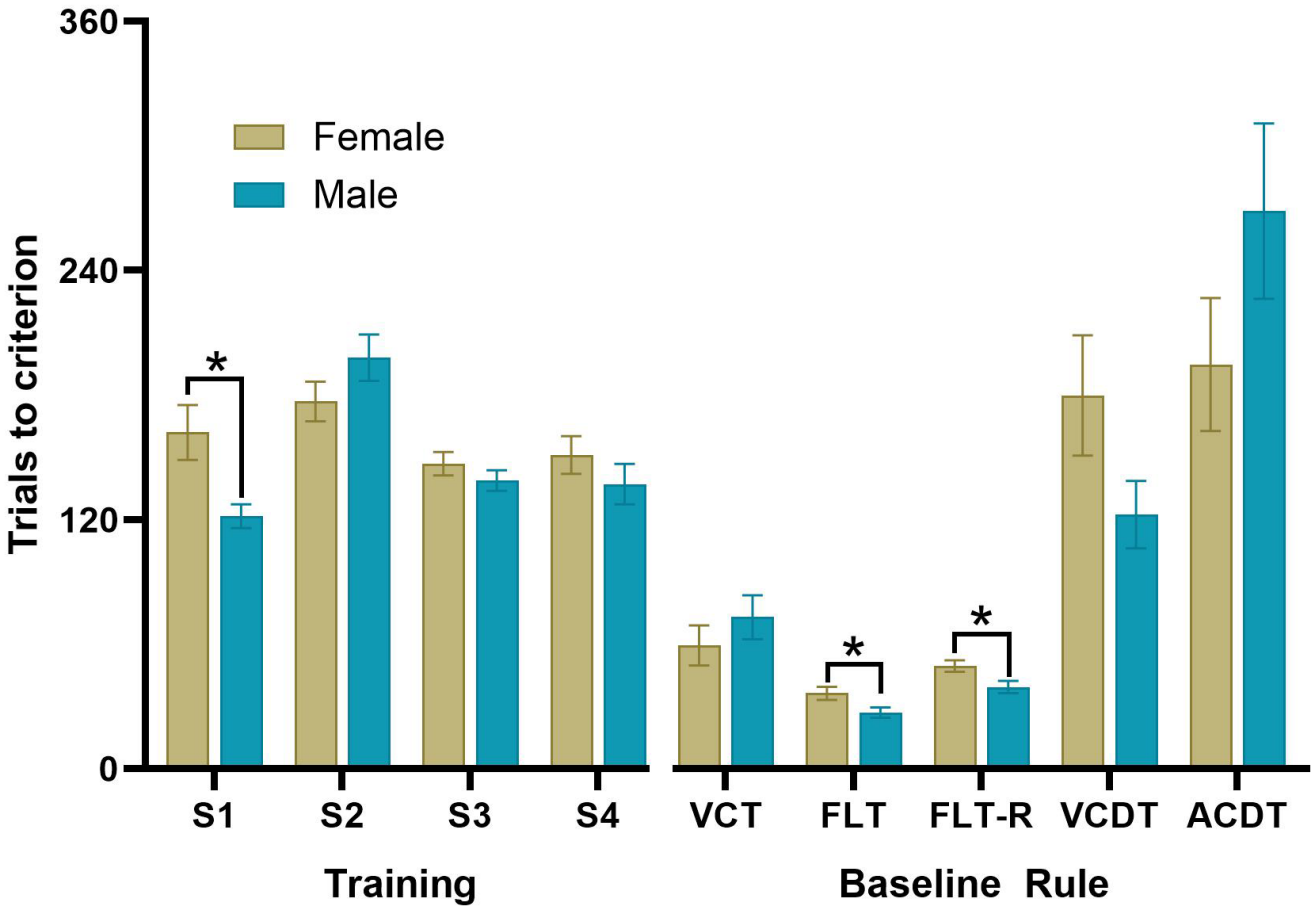
The average number of trials to criterion for males and females during the DSST. Measures of the average number of trials to criterion at baseline (prior to treatment with ketamine) at each stage of the DSST consisting of the VCT, FLT, FLT-reversal, VCDT and ACDT, *p<0.05 (n=42 females, n=42 males).

We trained and tested 84 rats using the optimised DSST protocol. The average number of sessions required for instrumental training was 9. At baseline, rats took an average of 4 sessions to complete 5 shifts in strategy (**Figure 5**). There was no significant difference between Cohort 4 and 5 for the number of trials to criteria for each stage. During stage 1 of training males took significantly fewer trials than females to consume 100 pellets from the food magazine (*t*_(82)_=2.80*, p*=0.006, *d*=0.61). There were no Sex differences in the number of trials to reach criteria during training on Stages 2, 3 or 4. There was a significant effect of the rule on the number of trials to criteria (*F*_(4,82)_=35.64*, p*<0.001, η_p_^2^ = 0.30), with the VCDT and ACDT taking significantly more trials to complete than the FLT or FLT-R rules. Females took significantly more trials to complete the FLT (*t*_(82)_=2.31*, p*=0.023, *d*=0.51) and FLT-reversal rules (*t*_(82)_=2.45*, p*=0.016. *d*=0.54) compared to males.

### 3.4 Acute ketamine manipulation

**Figure 6:**
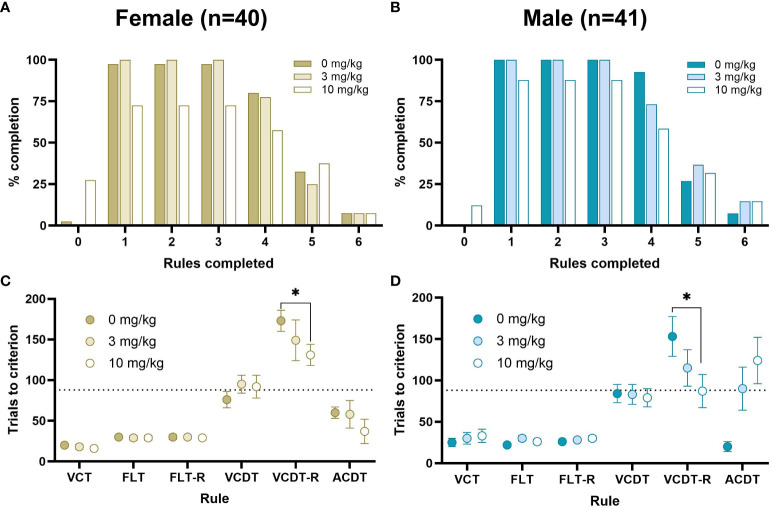
Percent completion and average number of trials to criterion in response to ketamine. The percentage of rats completing each stage is shown for females **(A)** and males **(B)**, Measures of the average number of trials to criterion are shown at each stage of the DSST consisting of the VCT, FLT, FLT-reversal, VCDT, VCDT-reversal and ACDT for females **(C)** and males **(D)** in response to 0, 3 or 10 mg/kg. The dotted line represents the average number of trials predicted to reach a CCR of 6 within 240 trials. There were no Sex x Dose interactions. Data for the VCT, FLT, FLT-reversal and VCDT rules were analysed using within-subjects ANOVA. Data for VCDT-reversal and ACDT rules were analysed with Cox regression to account for missing values; 10 mg/kg ketamine significantly improved performance compared to vehicle on the VCDT-reversal rule (HR=0.407, 95%CI=0.229–0.722, *p*=.002) *p<0.05.

**Figure 7:**
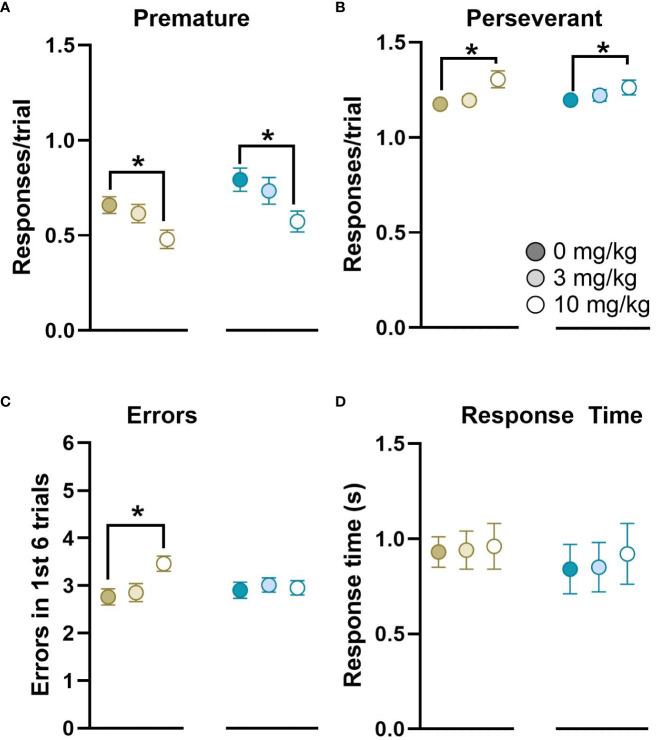
Overall effects of ketamine on responses on the DSST. Ketamine treatment reduced the number of premature responses **(A)** and increased the number of perseverant responses **(B)** to the centre nose poke used to initiate trials in both female and male rats. There was a significant increase in the number of errors in the first 6 trials after a rule change (for VCT, FLT, FLT-reversal and VCDT) for female, but not male rats **(C)**. There was no significant effect of ketamine on average response time **(D)**. Within each panel females are shown on the left, and males on the right in response to 0, 3 or 10 mg/kg ketamine. n=40 females and n=41 males, *p<0.05.

Following successful completion of 5 strategy shifts rats were treated with a dose response (0, 3, 10 mg/kg) of ketamine over 3 consecutive days. Rats administered 10 mg/kg were more likely to fail (16/81) to complete any rules than rats administered 0 (1/81) or 3 mg/kg (0/81). There were no significant effects of Day on the number of trials to criterion for each stage. There was a significant effect of Cohort on the acquisition of the FLT rule (*t*_(82)_=3.30*, p*<0.001, *d*=0.75, Cohort 1: 31.70 ± 1.51, Cohort 2: 22.50 ± 1.30, mean ± SEM). However, there were no significant interactions between Cohort x Sex or Cohort x Ketamine, and so all data were pooled for Cohort and Testing Day to examine the effects of Ketamine and Sex. There were no significant effects of Sex, or Sex x Ketamine on the number of stages completed (**Figure 6**). There was no significant effect of ketamine on the trials to criterion for the VCT, FLT or FLT-reversal rules based on a within-subject ANOVA. The majority of rats were unable to pass the VCDT-reversal, ACDT or ACDT-reversal rules, and this was unbalanced in the Latin-square design. We used Cox regression analysis to calculate HR with 95% CIs to compare the effects of ketamine on the number of trials to criterion for the VCDT-reversal rule, which revealed that there was an improvement in performance following treatment with 10 mg/kg ketamine (HR=0.407, 95%CI=0.229–0.722, *p*=0.002). By contrast, there was no significant effect of either 3 mg/kg (HR=1.38, 95%CI=0.52–3.64, *p*=0.52) or 10 mg/kg (HR=2.38, 95%CI=0.64–8.86, *p*=0.20) ketamine on the ACDT rule. There was insufficient data to analyse the response to the ACDT-reversal rule. Overall, there were significant effects of ketamine on the number of premature and perseverant responses to the centre nose poke (used to initiate trials, *F*_(2,62)_=15.47*, p*<0.001, η_p_^2^ = 0.31, **Figure 7**). There was a significant effect of ketamine on the number of errors in the first 6 trials after a strategy shift in female, but not male, rats (Dose x Sex interaction *F*_(2,68)_=3.34*, p*<0.05, η_p_^2^ = 0.09). There was no significant effect of ketamine on response time.

## 4 Discussion

This study addressed multiple aims in designing and optimising an automated DSST and provided an overview of how rats perform on the shift from responding to a visual cue (VCT) to a fixed location (FLT). Several limitations found in previous protocol designs were addressed, resulting in reduced training time, reduced omissions, and automated strategy shifts within a single session. Importantly, the optimised DSST replicates key features of human cognitive flexibility tasks by requiring rodents to shift strategy, reverse a previously learned rule, update information, and inhibit previously rewarded responses within a single session. Finally, we were able to show in 2 separate cohorts of rats (40 males and 41 females) that they can be trained and tested using a within-subject Latin-square design for ketamine within 14 days.

Optimising the training time required to learn the DSST ensures that the task is high-throughput and useful to researchers performing behaviour on many rats. In the first experiment (Image discrimination), rats were trained to discriminate between separate images widely used in rodent touchscreen operant tasks, presented on a single screen. Rats trained on this protocol were unable to complete the protocol in fewer than 59 sessions compared to the simplified lever-press training whereby rats completed the training in fewer than 10 sessions. In the second experiment (dynamic reversal) we demonstrated that rats are able to make multiple within-session strategy shifts, and this was included in the optimised DSST. These data indicate that complex visual stimuli, while more ethologically similar to the CANTAB ID/ED task, were too complex for Sprague Dawley rats to learn quickly. Therefore, it was concluded that the final protocol should incorporate strategy shifts that differ across stimulus dimensions using a non-spatial central cue light or tone to increase task complexity. To do this, the VCDT and an ACDT were added. In each of these rules, the presence of a signal (cue or tone) indicates the correct response required, similar to the previously published signal detection task used in Sprague Dawley rats (44).

Key design features in the optimised DSST improve upon current cognitive flexibility tasks in rodents. For example, the requirement for self-initiation of trials ensured that omissions were low, reducing the ambiguity in interpreting results during lesion and pharmacological manipulations. Furthermore, the inclusion of automated rule-shifting rather than between-session shifting optimised the testing so that multiple rule shifts could be observed rapidly in a manner more relevant to human tasks. This improves the validity of interpreting flexible behaviour as between-session paradigms introduce the potential of long-term memory formation as being a confounding variable. Rats were required to initiate each trial with a nose poke to an illuminated central aperture so that rats would have increased engagement with the task, and also to provide a general measure of attention and reaction time throughout the session. The near absence of omissions is an improvement on previous protocols and is significant because omissions may be an indication of reduced sustained attention, motoric deficits, motivation, and other behavioural changes that are not readily interpretable and are not translatable to human behaviour on cognitive flexibility tasks (44).

NMDAR antagonists have been used for their predictive validity in modelling some of the positive symptoms of schizophrenia such as hyperlocomotion and negative symptoms that include social interaction deficits (45). Prior research has shown mixed findings of the effects of NMDAR antagonists depending on factors, such as the drug administered (46), cognitive flexibility assay used, and dosing schedule (39, 47, 48). In the current study, we found that 10 mg/kg ketamine altered behaviour across several different measures. First, ketamine had non-specific effects on premature and perseverant responses to the central nose poke to initiate each trial. Ketamine reduced premature responses and increased perseverant responses. However, ketamine did not impact the number of omissions, which has been reported on other tasks, such as the 5 choice-serial reaction time (5C-SRT) task (49). Omissions can occur in rodents on the 5C-SRT because they missed the stimulus presentation, but also because they were performing a competing behaviour, such as grooming. Moreover, omission rates typically increase on the 5C-SRT with drug administration (50).

We also showed that 10 mg/kg ketamine impaired performance in a sub-group of rats, such that 20% of rats were unable to complete any rules, and this affected twice as many females (11/40) as males (5/41). For the remaining 80% of rats there was no significant effect of ketamine on the number of rules passed, or on the number of trials to criterion for VCT, FLT, FLT-R or VCDT. The majority of rats were unable to pass the more complex rules within a single session (VCDT-reversal, ACDT, ACDT-reversal). For those that did we showed a significant effect of 10 mg/kg ketamine on the number of trials to criterion for the VCDT-reversal rule. Female and male rats required fewer trials to complete the VCDT-reversal after ketamine, which was the opposite of what we expected. Due to the reduced sample size for the VCDT-reversal and ACDT rules these conclusions should be interpreted with caution. Future studies could consider adopting a more stringent criterion for these more complex rules, such as two consecutive sessions >80–85% correct prior to drug administration.

Previous research has shown that acute ketamine administration (10 or 20 mg/kg) 1–4 hours before testing disrupts ED set-shifting in rodents (46, 47, 51). There is an important effect of timing, as acute doses administered 24 hours before testing do not show this same deficit (47). Therefore, an important factor to consider is the duration of testing and the short and long-term effects of ketamine if testing is prolonged. In the current within-subjects design study we saw a pronounced deficit in males in the final stage of the task at 10 mg/kg compared to 0 mg/kg. Notably, both males and females required a lower number of trials to criterion on the second reversal of the task showing acute ketamine administration facilitated performance at this stage.

Limited research has compared how acute ketamine affects motivation on the task in males and females, with subanesthetic doses shown to decrease motivation and win-stay behaviour at 10 mg/kg on a probabilistic reversal learning task (52). However, subsequent studies have shown only an increase in negative feedback sensitivity and no detectable long-term positive or negative effects on cognitive flexibility (53). While a multitude of factors may influence the short and long-term effects of ketamine on cognitive flexibility, sex has not been investigated in depth. One potential factor is the clearance of the drug, influenced by body weight, amount, and distribution of adipose tissue. Sex differences in the activity of ketamine-metabolising cytochrome P450 enzymes may also affect the amount of ketamine reaching target areas of the brain (54, 55). Given that session times do not exceed 60 minutes in the current study, we would not expect this to be a significant factor; however, it is essential to consider the within-subjects design of the study and the potential long-term effects of ketamine on cognitive flexibility.

Furthermore, inconsistent findings regarding the effects of ketamine and the influence of sex introduce complexity in interpreting the present results. Although females have been shown to perform worse during the reversal component of ASST in other species (56), this finding is not observed in rats (30). A novel finding was that 10 mg/kg improved reversal learning (VCDT-reversal) in both male and female rats. One hypothesis is that ketamine may have a more significant impact on novelty processing in male rats compared to females, as repeated higher doses of ketamine have been shown to impair this process in males (57), although this has not been thoroughly investigated in females. Furthermore, previous research has demonstrated that ketamine exerts a biphasic effect on cognition (23). This dual-phase impact may influence the observed behavioural outcomes, particularly when considering metabolism differences between males and females (55).

One drawback of establishing a novel sequence of rules on the current tasks is that it limits the ability to make conclusions based on previous strategy shifting tasks. Unlike the ASST, the current task does not incorporate ID shifts and ED shifts as there are no exemplars of stimuli within the same category that shift at an ED stage. Instead, there is a shift to a novel category of stimuli that have not previously been experienced. Therefore, we made sure that the rats were able to acquire each rule (VCT, FLT, VCDT, ACDT) prior to treatment with ketamine to ensure that rats had experience of each rule once. Additionally, one of the major limitations of the current task is variability in the attrition rate at the most difficult stages of the task (ACDT, ACDT-reversal). Future studies may consider providing additional training sessions to ensure animals can reliably complete later stages to ensure reproducibility of the task.

In summary, while there are various factors influencing the effects of ketamine on cognitive flexibility, including potential sex differences, the current results suggest higher doses of ketamine have opposite effects on different aspects of the DSST. Previous research has shown inconsistent findings regarding the impact of ketamine and the role of sex in cognitive tasks, suggesting a complex interplay of factors. Further investigation, and exploring variables such as drug metabolism, brain physiology, and behavioural strategies are necessary to provide a comprehensive understanding of how ketamine affects cognitive flexibility and why these effects differ between males and females.

The DSST incorporates rules of differing complexity, and, on average, rats completed 17 rules during the ketamine experiment out of a potential maximum of 23 consecutive rules (5 in the first exposure and 3x6 in the second exposure). We showed that during the 1st exposure the rats were not performing at chance; either they required fewer (VCT, FLT, FLT-reversal) or more trials (VCDT, ACDT) to acquire the rule than by chance (88 trials). During the 2nd exposure (after vehicle injection) the rats performed better than chance for the VCT, FLT, FLT-reversal and ACDT rules, whereas the response to VCDT and ACDT-reversal was not different to chance (see **Supplementary Figure 6**). Furthermore, although 1 female and 1 male rat passed all 23 consecutive rules, demonstrating that it is possible for a rat (of either sex) to successfully complete the optimised DSST, (see **Supplementary Figure 7**) this performance would be expected by chance. This result will be important when considering cognitive enhancing drugs because there is the capacity for rats to show improved as well as impaired performance (i.e. better or worse than chance). In human studies, the primary measure of importance is trials to criterion or the number of errors before a rule change (58). In rats, additional secondary measures can provide information on processing speed, attention, motoric effects, and impulsivity, and give information about the strategy employed by the rat on the DSST. Dimensional analysis was used to look at each measure of the DSST and how they correlated to establish the primary measures. PCA was applied to reduce the complexity of analysing the large datasets generated by the DSST to increase the interpretability of results. While the 13 measures identified are useful in building a picture of how the rat alters its strategy, PCA revealed that many measures on the task were correlated (see **Supplementary Figure 4**). Inappropriate responses, correct and incorrect responses, trials to criterion, and win-stay/lose-shift were closely correlated to the principal component suggesting that overall, these measures explain most of the variance within the data. Other measures were less closely correlated to the principal component such as perseverative errors. Timed measures such as average trial duration and average response duration were correlated closely with the second principal component. Together these suggest that a fast-performing rat can be good or bad at performing the task. The number of training sessions was not correlated to either component. This may be because rats generally train in a similar number of sessions and therefore this is not predictive of any variance within the dataset. It is important to note that this analysis was conducted in healthy rats and that these factors may load together differently depending on the specific manipulation conducted using the DSST.

While no single task represents a pure measure of a cognitive process, the DSST is designed to measure the ability of rats to adapt behaviour that progressively increases cognitive demands. Unlike the ASST and CANTAB IDED task, the DSST does not measure attentional set-shifting and as such is best conceptualised as a progression of the strategy shifting task developed by Floresco and colleagues (36). However, unlike the Floresco task, rats in the current study were not exposed to stimulus lights during training and thus did not have a bias towards learning the VCT faster than the FLT. This may allow performance of subsequent shifts to rely more on the intrinsic demands of the task rather than an initial bias present in the task. Extending the design of the Floresco task, the DSST includes non-spatial rules; the VCDT using a central cue light and the ACDT with a tone. These additional rules ensure a stimulus category change (light to tone) and increases the complexity of the strategy shift to examine cognitive flexibility when task demand increases. Crucially, the shifts between these rules are automated and rats progress at an individual pace that has increased translational relevance to human tasks. The Sprague Dawley rats may be more adept at flexibly changing strategies or simply that because they are an outbred, non-pigmented strain, these rats find the FLT easier to learn. Further studies are needed to directly compare strains on the DSST to see whether training protocol, housing, or light cycle impacted trials to criterion. This information may be useful in deciding the optimal strain on the DSST to increase the speed of screening drugs to screen their effects on cognition.

While there is no consensus regarding the number of distinct executive functions required in different forms of flexibility such as cognitive flexibility, task switching, and attentional set-shifting, the DSST requires engagement in the core executive functions of inhibition, working memory, and cognitive flexibility in shifting between strategies (59). Therefore, this task may have sensitivity to several cognitive domains that can be investigated and scored depending on the level of detail and specific research question. We also explored if any single measure on the VCT could predict performance on the FLT. If we can predict high and low performance, this may give an indication of which core traits are necessary for behavioural flexibility. For the multiple linear regression model, the primary outcome variable was the number of trials to criterion. Out of the 13 outcome measures tested, the greatest predictor of trials to criterion was the number of lose-shift responses on the VCT. The number of incorrect trials was also a significant predictor of the trials to criterion on the FLT in addition to the average trial rate and a lesser extent, the number of win-stay responses.

Several useful conclusions can be drawn from the development and optimisation of the DSST. Firstly, stimulus-reward pairings using sequentially presented images is not optimal for task performance in Sprague Dawley rats and dual presentation may be required for optimal performance such as those used in touchscreen tasks (33). Although the presentation was of a spatially cueing image near the rewarded response lever, Sprague Dawley rats were unable to successfully acquire this association. Previous research suggests even in pigmented strains, 15–20 sessions are needed to acquire stimulus-response pairings and that there is a ceiling of around 75% accuracy while rats perform the task (60). Subsequent research has also suggested that increasing the time a rat can respond to visual stimuli can lead to a faster rate of learning (61). These perceptual constraints may be at odds with the goal of achieving a high throughput task, which was a key aim of the current study, therefore, alternative stimuli were selected.

Overall, the DSST has fewer training sessions compared to visual discrimination training, reduced omissions, and automatically switches between strategies. The performance of Sprague Dawley rats on the current task appears superior to Long Evans on similar strategy shifting tasks, although further testing with Long Evans on the DSST is required. Given the strategy shift to the FLT is easier for this strain to complete, future studies should incorporate the VCDT and ACDT as well as reversals to increase the difficulty and avoid ceiling effects. We were also able to show that acute low doses of ketamine differentially affect performance (unaltered, impaired or enhanced) and this is dependent on the strategy and sex. The DSST has potential utility in advancing our understanding of cognitive processes and the underlying neurobiological mechanisms related to flexibility in neuropsychiatric and neurological conditions where executive dysfunctions occur. Additionally, the ability to measure attention, response inhibition, and working memory make the task particularly suited to assessing key domains affected in schizophrenia. Crucially, this study highlights that the DSST emulates key features of human tasks and has some advantages over similar automated rodent tasks to be used as a high throughput cognitive flexibility assay in rodents.

## Data availability statement

The raw data supporting the conclusions of this article will be made available by the authors, without undue reservation.

## Ethics statement

The animal study was approved by The University of Queensland Animal Ethics Committee. The study was conducted in accordance with the local legislation and institutional requirements.

## Author contributions

JF: Conceptualization, Data curation, Formal analysis, Investigation, Methodology, Visualization, Writing – original draft, Writing – review & editing. SA: Data curation, Software, Validation, Writing – review & editing. JK: Conceptualization, Supervision, Writing – review & editing. TB: Conceptualization, Funding acquisition, Methodology, Project administration, Resources, Supervision, Writing – original draft, Writing – review & editing.
