# Supplementary material for: The dynamic strategy shifting task: Optimisation of an operant task for assessing cognitive flexibility in rats
Source: Front Psychiatry. 2024 Jun 28;15:1303728. doi: 10.3389/fpsyt.2024.1303728 (PMC11240049; doi:10.3389/fpsyt.2024.1303728)
Supplement: Supplementary file 1 [file DataSheet_1.docx]

**Supplementary methods and results**

*Consecutive correct response manipulation*

The next manipulation implemented a consecutive correct response (CCR) count to automate shifting between the VCT and FLT. Rats were tested over 6 days to observe how many shifts they could make when a CCR count of 8 was required. After 6 days an incrementing CCR count was trialled whereby rats reversed between strategies beginning at CCR count of 3. This then incremented such that the rat required consecutively more CCR count before a strategy shift occurred (up to a maximum of 8 CCR count in a row).

*Computer simulation versus rat performance*

A computer simulation was run 84 times for at least 1200 trials, by selecting either 0 or 1 randomly and running a counter to look for consecutive 1s (CCR=6). When CCR=6 the counter reset until the next CCR=6. For the initial 5 rules (1st exposure) there were unlimited trials. For the next 7 rules (2nd exposure), to progress to the next rule a CCR=6 must be completed within 240 trials (this was set at 120 trials in session 1 (am), otherwise the trial ends, the rats then received a second session (pm) and had 120 trials to complete a rule, which gave a maximum of 240 trials to count the number of rules completed). The average number of trials to complete (TTC) rules in the first exposure with no limit is 126, and this is reduced to 88 when the maximum number of trials is reduced to 240. Data were compared to chance by *t*-test.

*Statistical analysis*

Principal components analysis (PCA) was used to explore the explanatory power of responses on VCT and FLT rules. Correlational analysis between outcome measures obtained during the acquisition of the VCT and the number of trials to criterion on the FLT was conducted using multiple linear regression. The number of trials to criterion were used as the dependent variable with correct trials, incorrect trials, inappropriate responses, win-stay, lose-shift, average trial duration, average response duration, and average trial rate as the independent variables.

*Consecutive correct response manipulation*

Over 6 days of implementation of an automated CCR counter, rats achieved a mean number of 5.65 (±0.48) switches on the first day and 1.98 (±0.21) on the final day with no significant difference between males and females observed (*t*_(15)_=0.82*, p*=0.44, *d*=-0.11) (Supp. Fig. 1A. No effect of Sex was indicated on the number of sessions to criterion (*F*_(1,10)_=2.77*, p*=0.10, η_p_^2^=0.59) (Supp. Fig. 1B).

*Performance on response outcome measures on the DSST*

A paired samples t-test showed a significant decrease in % accuracy on the FLT compared with the VCT (*t*_(5)_=11.77*, p*<0.0001, *d*=2.88) (Supp. Fig. 3A). Rats had a significantly greater average trial duration on the FLT (*t*_(4)_=9.962*, p*<0.001, *d*=-1.39) (Supp. Fig. 6B) and made significantly more premature head entries (*t*_(4)_=10.10*, p*<0.001, *d*=-0.91) (Supp. Fig. 6C) errors, but perseverative head entries were unchanged (*t*_(5)_=1.702*, p*=0.150, *d*=-0.31) (Supp. Fig. 6D). A two-way ANOVA indicated a significant effect of Stage on win-stay/lose-shift strategy (*F*_(3,106)_ =3.54*, p*=0.02, η_p_^2^= 0.20) (Supp. Fig. 6E) with multiple comparisons analysis indicating that rats used a lose-shift strategy more on the FLT (*p*=0.013). Rats made no omissions on the VCT or the FLT.

*Principal components analysis*

To explore the relationship between outcome measures of the task, a principal component analysis was conducted. This multivariate technique was employed to explore how intercorrelated each outcome measure in the task is to observe redundant measures that need not be investigated in further experiments and simplify future analysis. There were two main clusters of measures identified (Supp. Fig. 4). The first cluster consisted of the following measures: the number of Incorrect Responses, Inappropriate Responses, Trials to Criterion, and Correct Trials and the percentage of Win-stay/Lose-shift Responses. The second cluster consists of Perseverative Errors, Lose-shift Responses, and Average Trial Duration. Multiple linear regression analysis was performed to examine the relationship between the outcome measures during the VCT and their correlation with the number of Trials to Criterion during the FLT. In this analysis, the predictor variables were the number of Training Sessions, Incorrect Responses, Average Response Duration, Average Trial Duration, Average Trial Rate, and percentage of Win-stay/Lose-shift Responses. A significant regression equation was indicated (*F*_(9,21)_=3.48*, p*=0.009) with an R^2^ of 0.77. Measures associated with the number of Trials to Criterion in the FLT was equal to 79.57 -1.15 (Training Sessions) + 1.65 (Incorrect Trials) + 9.54 (Average Response Duration) -0.67 (Average Trial Duration) -4.23 (Average Trial Rate) +.48 (Win-stay Responses) -2.23 (Lose-shift Responses). Significant predictors of the number of Trials to Criterion required in the FLT from the data in the VCT included the Incorrect Trials (*p*=0.021), Average Trial Rate (*p*=0.039), and Lose-Shift Responses (*p*=0.007) (Supp. Table 1).

*Response to non-spatial rules on the DSST*

A two-way ANOVA indicated there was an effect of Stage on Trials to Criterion (*F*_(4,93)_=40.16*, p*<0.001, η_p_^2^=0.59) . Post-hoc analyses were performed to compare each stage to the previous stage to examine the level of difficulty required to achieve a CCR count of ten. Multiple comparisons with Bonferroni correction indicated no significant increase in the number of trials to criterion between the VCT and FLT (*p*>0.999), but a significant increase in the number of trials to criterion required shifting from the FLT to the VCDT (*p*<0.001), VCDT to the ACDT (*p*<0.001), and the ACDT to the ACDT reversal (*p*<0.001) (see Supp. Fig. 5).

Supplementary Figure legends

**Supplementary Figure 1.** Graphical flow chart of code used during the DSST protocol to show how the protocol was divided into distinct stages for the visual cue task (VCT), fixed location task (FLT), visual continuous detection task (VCDT) and auditory continuous detection task (ACDT). The underlying code was written using MedState notation.

**Supplementary Figure 2.** **Consecutive correct response manipulation.** A) Presents the CCR manipulation over 6 days of dynamic reversals. In this manipulation, male and female (n=8 males and n=8 females) rats were required to achieve an increasing number of CCRs beginning at 3 to initiate a reversal. B) Shows the number of sessions to criterion.

**Supplementary Figure 3. Performance on response outcome measures on the DSST.** A) The trials to criterion for the VCT and FLT with the automated shift within session. B) Correct trials made on the VCT and FLT. C) Incorrect trials made on the VCT and FLT. D) The total inappropriate responses made by head entry (HE) in the central nose poke and central reward receptacle. E) The perseverative errors made when rats switched from the VCT to the FLT. Mean ± S.E.M shown. ****p*<0.001, *****p*<0.0001 (n=8 males and n=8 females).

**Supplementary Figure 4. PCA Analysis.** Component Plot showing each outcome measure and its relationship to components 1 and 2.

**Supplementary Figure 5. The average number of trials to criterion during optimisation of the DSST.** Stages shown include the VCT (n=34), FLT(n=33), VCDT(n=27), ACDT(n=24), and ACDT reversal(n=13). ***p*<0.01, ****p*<0.001.

**Supplementary Figure 6.** Comparison between performance of rats and by computer simulation. A. The upper panel shows the percent completion from rats (12 rules comprising 5 from the 1st exposure, and 7 from the 2nd exposure after they received 0 mg/kg), and this is compared with a computer simulation (RANDOM number between 0 and 1) run 84 times for at least 1200 trials. B. The lower panel shows the number of trails to criterion compared with chance (128 for the 1^st^ exposure and 88 for the 2^nd^ exposure). * *p*<0.05.

**Supplementary Figure 67** Total number of rules completed by rats used in the ketamine experiment (n=40 females, n=41 males). Two rats completed 23 consecutive rules (5 prior to drug treatment and then 6 rules for each of 3 treatment days), female #44, and male #12 showing their trials to completion for each rule at each stage.

| Measure | Component 1 | Component 2 |
| --- | --- | --- |
| Innapropriate responses | 0.977 | 0.131 |
| Trials to criterion | 0.956 | 0.169 |
| Lose-shift | 0.889 | 0.169 |
| Incorrect | 0.879 | -0.008 |
| Correct | 0.847 | 0.227 |
| Perseverative errors | 0.724 | -0.173 |
| Win-stay | 0.634 | 0.222 |
| Training sessions | -0.183 | 0.123 |
| Average trial rate | 0.093 | -0.896 |
| Average trial duration | 0.366 | 0.862 |
| Average response Duration | 0.049 | 0.848 |

**Supplementary Table 1 Rotated component matrix of outcome measures**
