# Supplementary figures and images for: The dynamic strategy shifting task: Optimisation of an operant task for assessing cognitive flexibility in rats
Source: Front Psychiatry. 2024 Jun 28;15:1303728. doi: 10.3389/fpsyt.2024.1303728 (PMC11240049; doi:10.3389/fpsyt.2024.1303728)

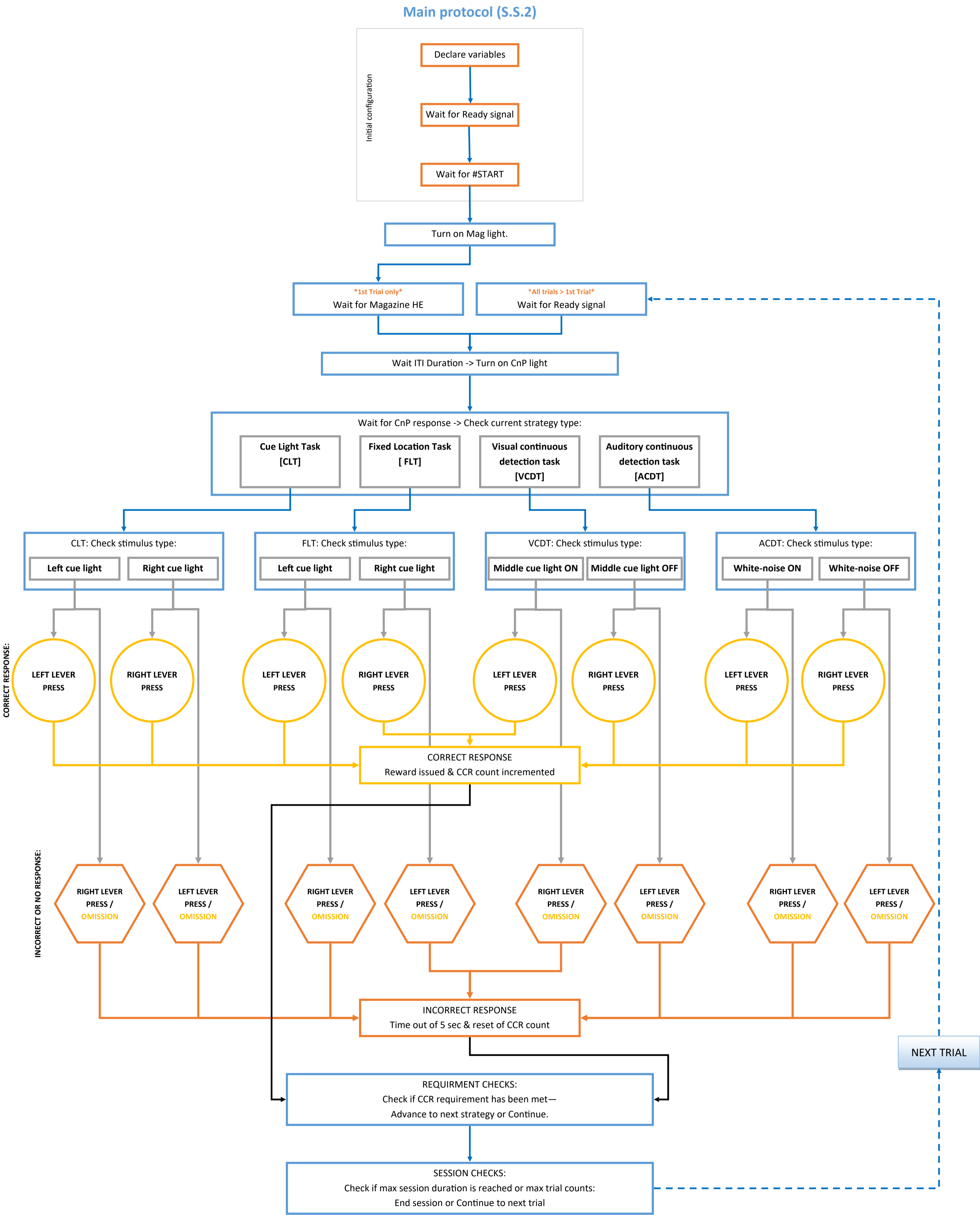

Supplement: Supplementary file 2 [file Image_1.pdf]

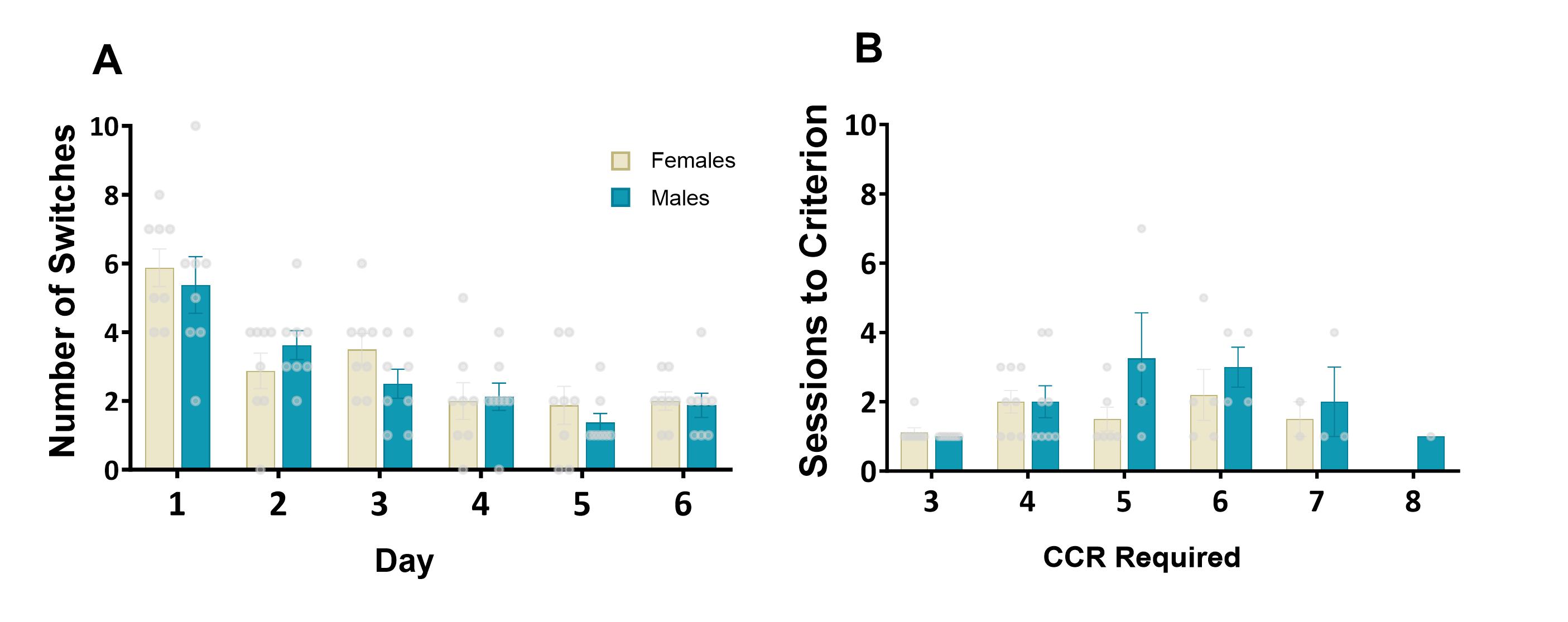

Supplement: Supplementary file 3 [file Image_2.jpeg]

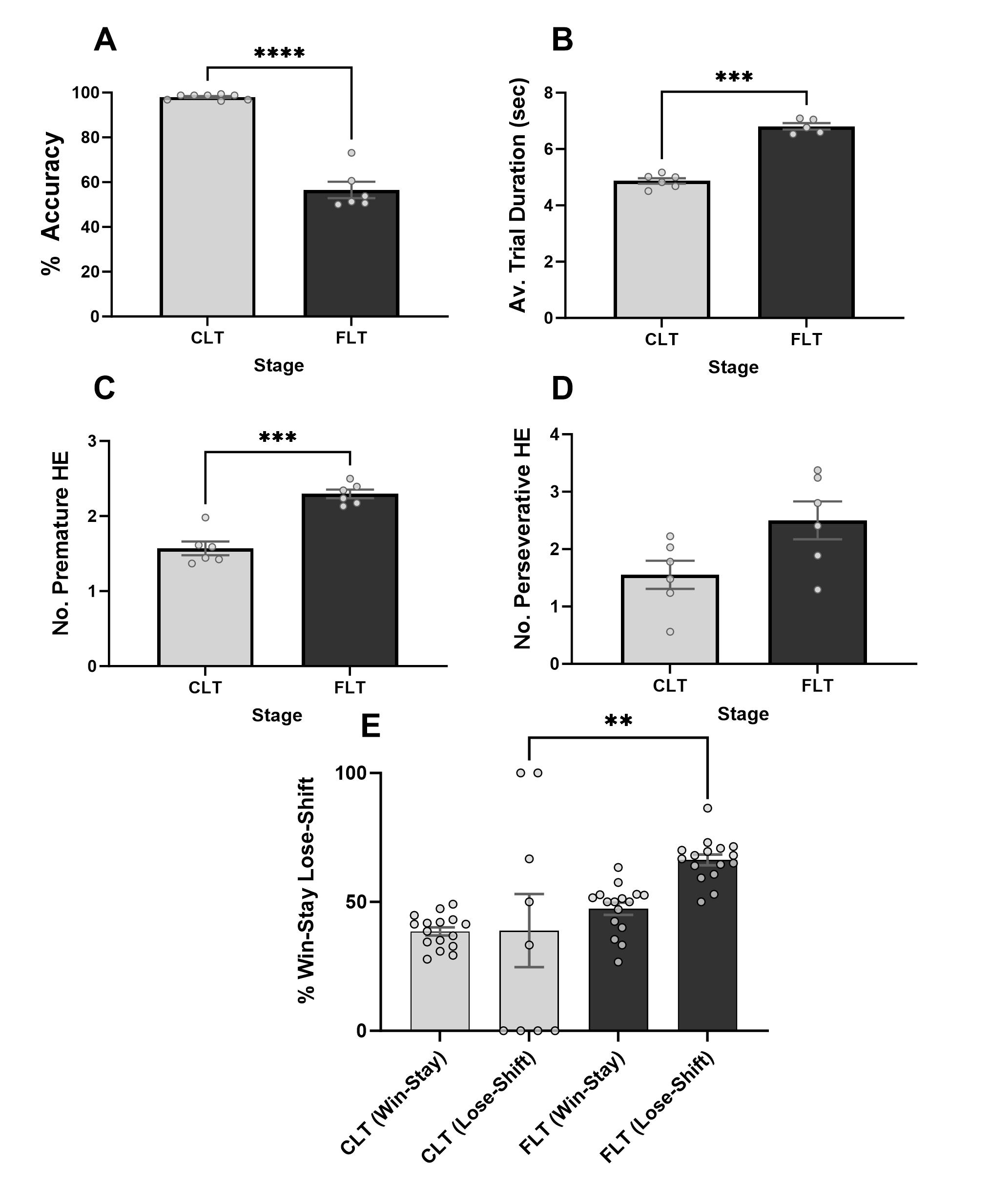

Supplement: Supplementary file 4 [file Image_3.jpeg]

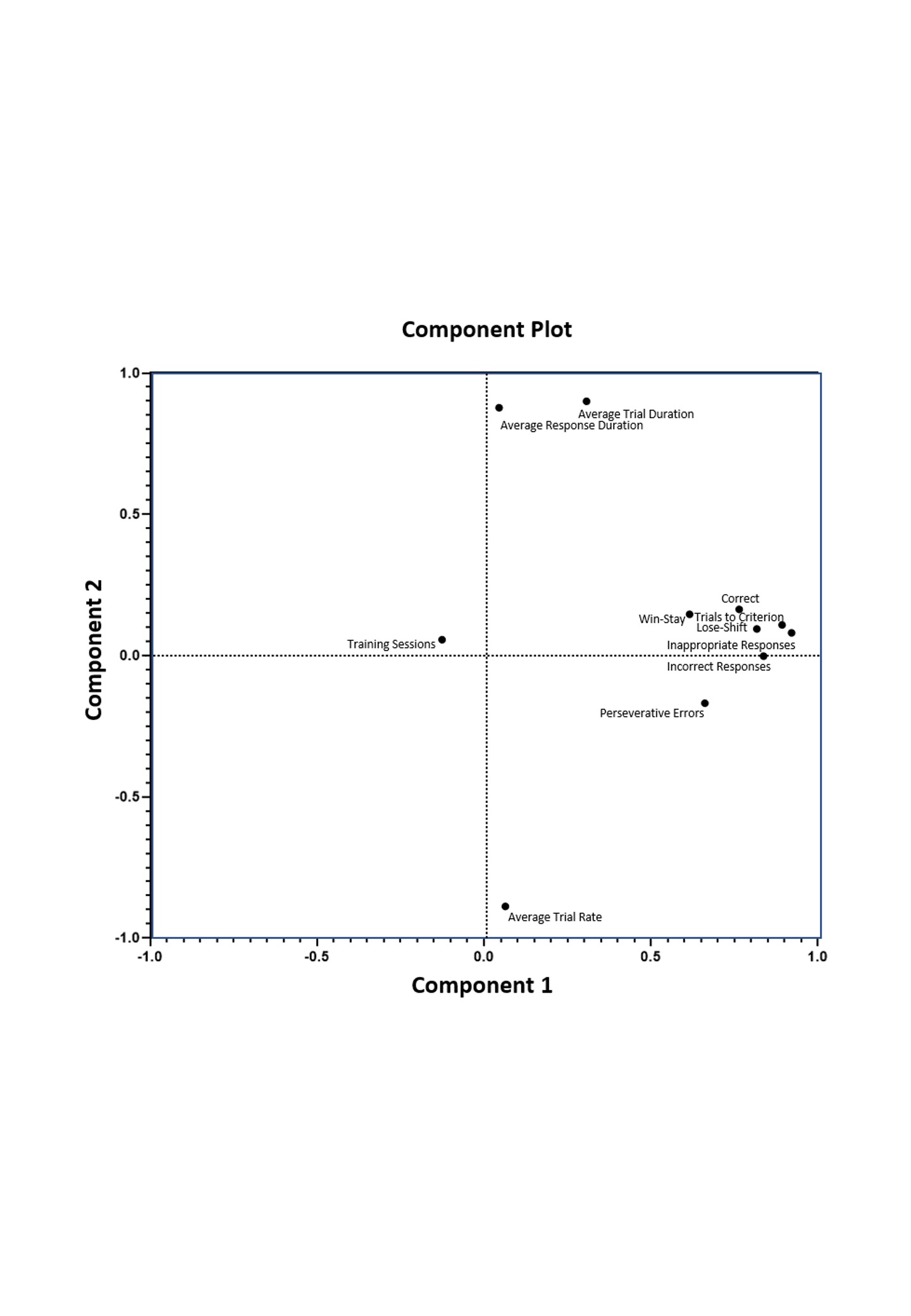

Supplement: Supplementary file 5 [file Image_4.jpeg]

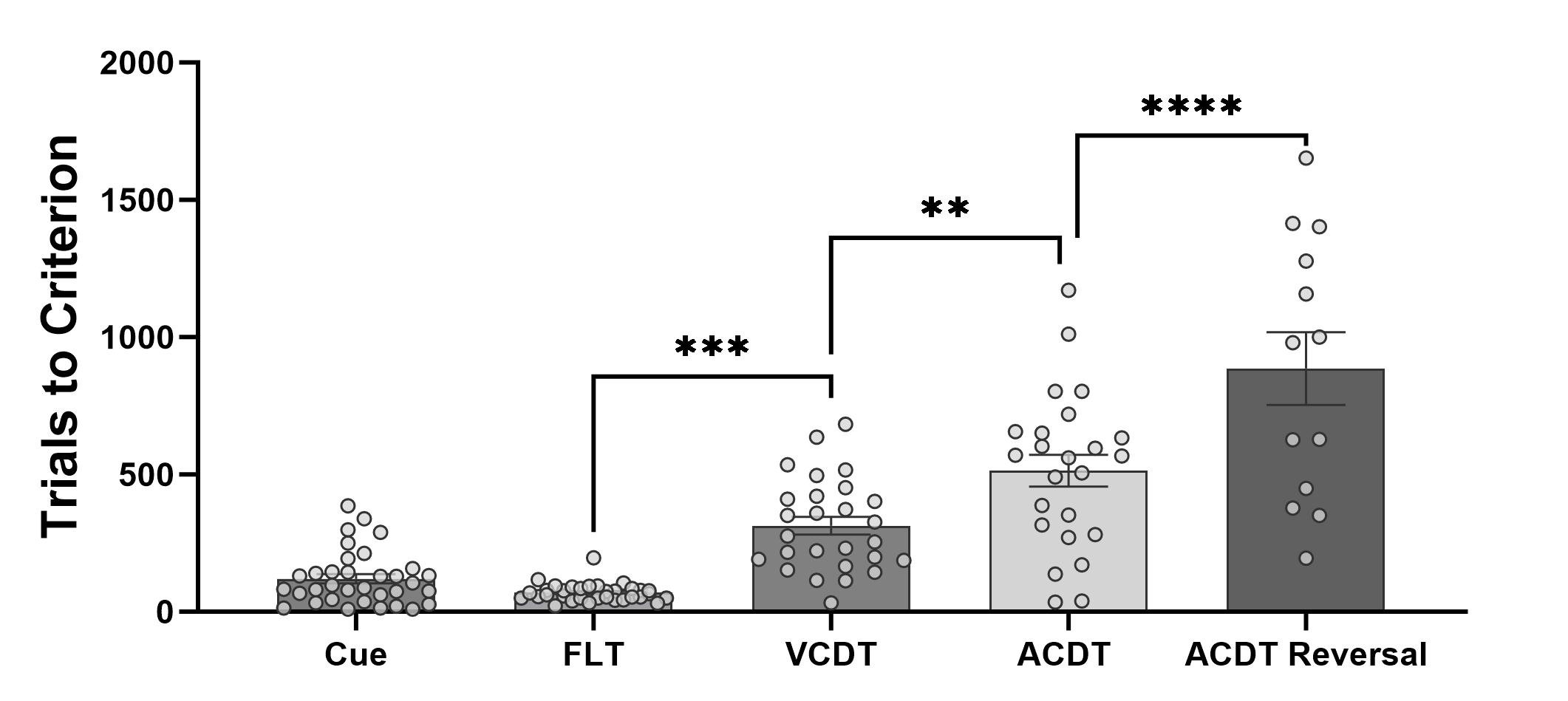

Supplement: Supplementary file 6 [file Image_5.jpeg]

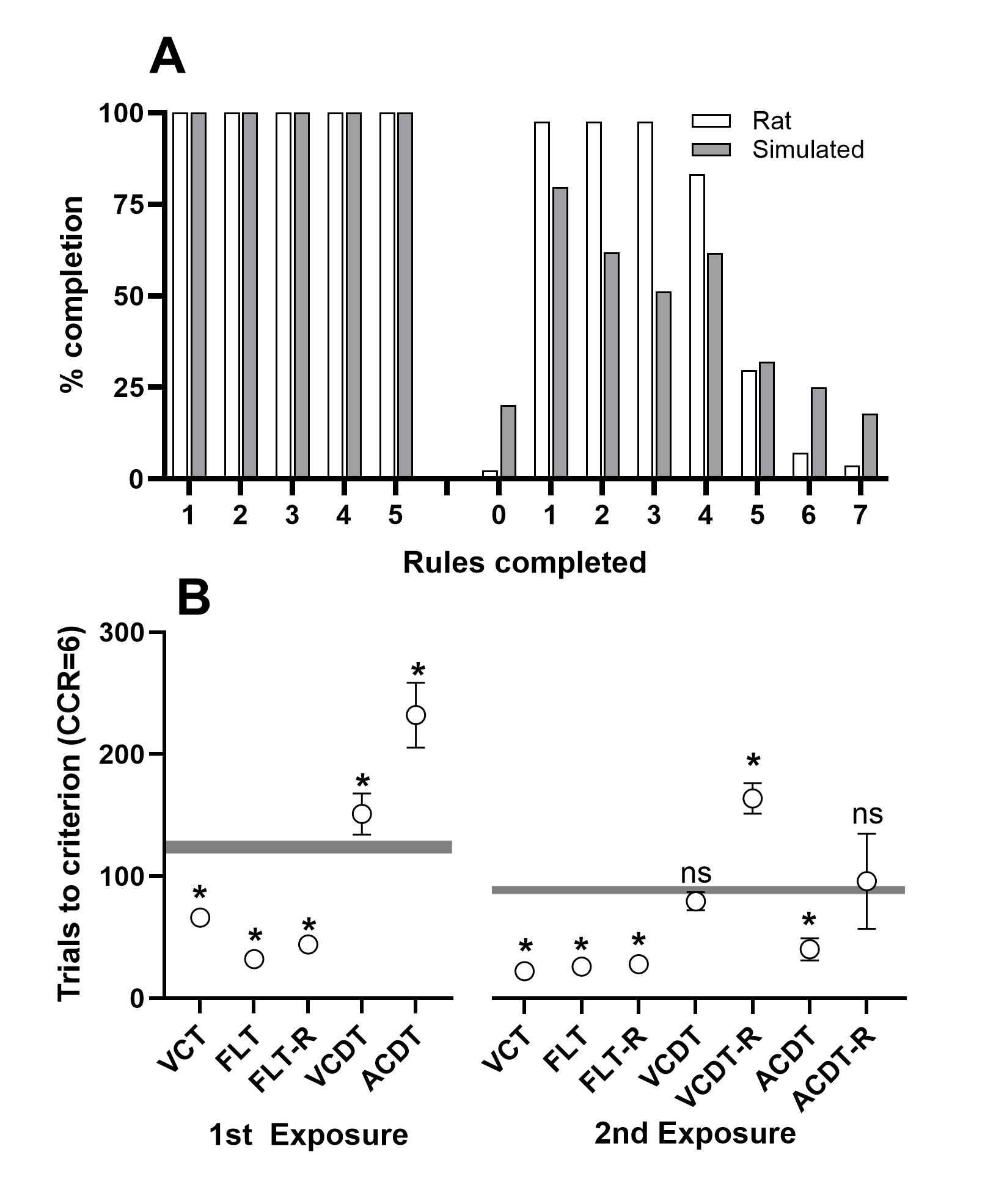

Supplement: Supplementary file 7 [file Image_6.jpeg]

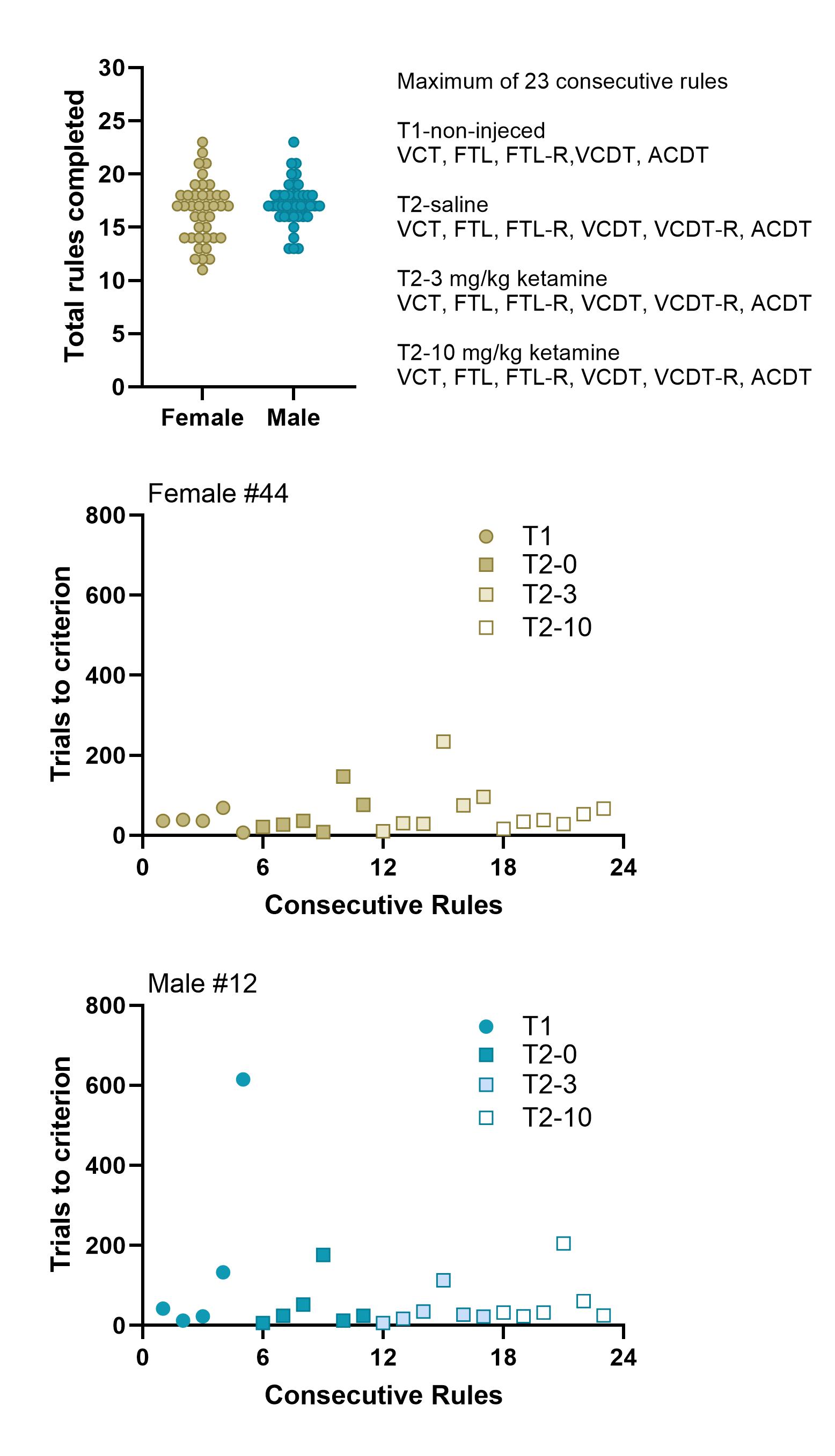

Supplement: Supplementary file 8 [file Image_7.jpeg]
